# Potential role for PADI-mediated histone citrullination in preimplantation development

**DOI:** 10.1186/1471-213X-12-19

**Published:** 2012-06-19

**Authors:** Rui Kan, Mei Jin, Venkataraman Subramanian, Corey P Causey, Paul R Thompson, Scott A Coonrod

**Affiliations:** 1 Baker Institute for Animal Health, Cornell University, Ithaca, NY, 14850, USA; 2 Department of Chemistry, The Scripps Research Institute, 130 Scripps Way, Jupiter, FL, 33458, USA; 3 Department of Chemistry, University of North Florida, Jacksonville, FL, 32224, USA

**Keywords:** Peptidylarginine deiminase, Citrullination, Histone modification, Preimplantation development, Cl-amidine

## Abstract

**Background:**

The peptidylarginine deiminases (PADIs) convert positively charged arginine residues to neutrally charged citrulline on protein substrates in a process that is known as citrullination or deimination. Previous reports have documented roles for histone citrullination in chromatin remodeling and gene regulation in several tissue types, however, a potential role for histone citrullination in chromatin-based activities during early embryogenesis has not been investigated.

**Results:**

In the present study, we tested by laser scanning confocal indirect immunofluorescence microscopy whether specific arginine residues on the histone H3 and H4 N-terminal tails (H4R3, H3R2 + 8 + 17, and H3R26) were citrullinated in mouse oocytes and preimplantation embryos. Results showed that all of the tested residues were deiminated with each site showing a unique localization pattern during early development. Given these findings, we next tested whether inhibition of PADI activity using the PADI-specific inhibitor, Cl-amidine, may affect embryonic development. We found that treatment of pronuclear stage zygotes with Cl-amidine reduces both histone H3 and H4 tail citrullination and also potently blocks early cleavage divisions *in vitro*. Additionally, we found that the Cl-amidine treatment reduces acetylation at histone H3K9, H3K18, and H4K5 while having no apparent effect on the repressive histone H3K9 dimethylation modification. Lastly, we found that treatment of zygotes with trichostatin A (TSA) to induce hyperacetylation also resulted in an increase in histone citrullination at H3R2 + 8 + 17.

**Conclusions:**

Given the observed effects of Cl-amidine on embryonic development and the well documented correlation between histone acetylation and transcriptional activation, our findings suggest that histone citrullination may play an important role in facilitating gene expression in early embryos by creating a chromatin environment that is permissive for histone acetylation.

## Background

The fundamental repeating unit of chromatin is the nucleosome that contains two superhelical turns of DNA wrapped around an octamer of two copies each of the core histones H2A, H2B, H3 and H4 [1,2]. Resolution of the nucleosome structure revealed that the N-terminal histone tails protrude from the nucleosomal core in an unstructured manner [3] and contain an ever-growing number of post-translational modifications such as acetylation, methylation, phosphorylation, and more recently, citrullination [4,5]. Importantly, these modifications, or “marks”, play critical roles in many cellular functions, including DNA replication, condensation, and repair, as well as gene regulation [6]. Of these modifications, histone acetylation is perhaps most strongly associated with gene regulation. Increasing levels of histone acetylation are correlated with a transcriptionally permissive state whereas deacetylated histone are closely associated with transcriptional repression [7]. Histone acetylation is also implicated in the activation of embryonic gene expression in preimplantation embryos [8]. For example, previous reports investigating late two-cell embryos have found that inducing histone hyperacetylation with HDAC inhibitors stimulates global transcription [9] and depletion of HDAC1 by RNAi results in elevated levels of specific gene targets [8]. Previous studies in somatic cells have demonstrated that specific histone modifications can directly affect the levels of other marks and this interplay leads to a complex mechanism of gene regulation, frequently referred to as the “histone code” [10,11]. While fewer of these types of studies have been carried out in early embryos, several reports have found that cross-talk exist between histone acetylation and histone methylation in normal and cloned embryos [12].

PADI enzymes are increasingly being associated with the regulation of chromatin structure and gene activity via histone citrullination. For example, we have found that PADI4-mediated citrullination of histone H4 arginine 3 at the *TFF1*promoter in MCF7 cells appears to regulate the expression of this canonical estrogen receptor target [13]. Others have shown that PADI4-mediated histone citrullination plays a role in regulating other target genes such as *TRP53* and *OKL38*[14,15]. In addition to PADI4, we recently found that PADI2 localizes to the nucleus of mammary epithelial cells and appears to target histone H3 for citrullination [16], thus suggesting that multiple PADIs regulate chromatin-based activities.

We have previously documented that oocyte -and-embryo abundant PADI6 is required for female fertility, with PADI6-null embryos arresting at the two-cell stage of development [17]. Given the growing body of literature linking PADI enzymes to histone citrullination and the abundance of PADI6 in oocytes and early embryos, we first tested whether histones were citrullinated in oocytes and preimplantation embryos. Next, we treated embryos with the PADI-specific inhibitor, Cl-amidine, to confirm that the observed citrulline marks were generated by PADI activity and also to test whether inhibition of PADI activity may affect preimplantation development *in vitro*. Lastly, to gain insight into potential mechanisms by which histone citrullination may regulate gene activity, we tested whether inhibition of PADI activity in early embryos affects histone acetylation and whether induction of histone hypoacetylation affected levels of histone citrullination. Findings from this study are discussed below.

## Results and discussion

### Citrullination of histone H3 and H4 tails in oocytes and in preimplantation embryos appears to be robust and dynamic

**Figure 1:**
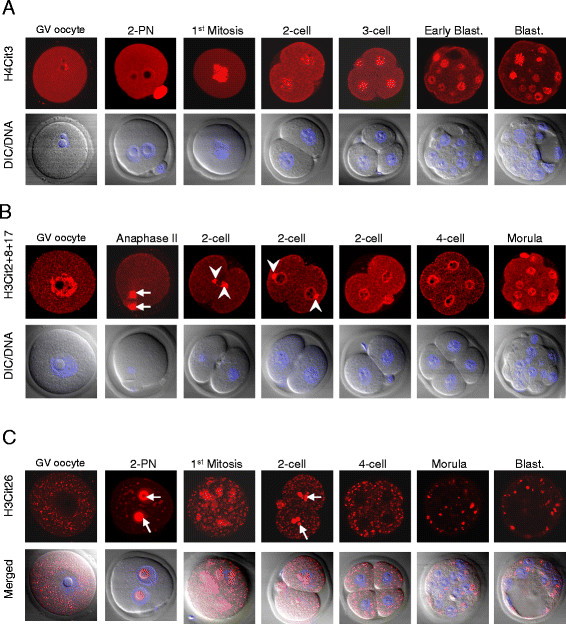
**Temporal and spatial distribution of histone H3 and H4 citrullination in oocytes and preimplantation embryos. A.** Oocytes and embryos were isolated from CD1 female mice, fixed, permeabilized, and immunolabeled with antibodies against H4Cit3 (red). **B.** Same as (A) except anti-H3Cit2 + 8 + 17 antibodies (red) were used. Spindle apparatus and microtubule based mid-bodies are indicated by arrows and arrowheads, respectively. **C.** Same as (A) except anti-H3Cit26 antibodies (red) were used. Nucleoli are denoted by arrows. All samples were counterstained with 4′,6-diamidino-2-phyenylindole (DAPI) to visualize DNA (Blue). Cells were imaged by laser scanning confocal microscopy. DIC, differential interference contrast. PN, pronuclear. Blast., blastocyst.

In the present study, the status of histone H3 and H4 citrullination during early development was investigated by staining fully grown mouse germinal vesicle (GV) stage oocytes and preimplantation embryos with three site-specific citrullinated histone antibodies: anti-histone H4 citrulline 3 (H4Cit3), anti-histone H3 citrulline 2, 8, and 17 (H3Cit2 + 8 + 17) and anti-histone H3 citrulline 26 (H3Cit26) [13,16,18]. Results from these confocal indirect immunofluorescence studies showed that H4Cit3 staining was observed in the nucleus and cytoplasm of oocytes and early embryos at interphase. Interestingly, the strongest staining for this citrulline modification appears to occur on mitotic metaphase chromatin, suggesting that H4Cit3 may play a role in chromatin condensation or decondensation at metaphase (Figure 1A). Staining with the anti-H3Cit2 + 8 + 17 antibody found that this modification also stained nuclei during interphase (Figure 1B). However, at metaphase, this antibody appeared to primarily stain the spindle apparatus (Figure 1B; arrows in the panel of anaphase II) and a structure at opposed regions of two cell blastomeres that appears to be the microtubule-containing mid-body [19,20] (Figure 1B; arrowheads in panels of 2-cell). Interestingly, in somatic cells, an anti-phospho-H3 serine 10 antibody detected histones with this modification at the anaphase spindle apparatus and at the mid-body during cytokinesis [21]. These investigators speculated that this modification may mark histones for removal from the nucleosome or possibly that this modified histone may actually play a direct role in cytokinesis by functioning at the mid-body [22]. It is currently not known whether citrullinated histones in the cytoplasm of oocytes and early embryos might have a similar function. Alternatively, however, it is also possible that the observed spindle and mid-body staining with the H3Cit2 + 8 + 17 antibody is non-specific in nature.

The staining pattern observed with the anti-H3Cit26 was perhaps the most interesting of all. Results found that both oocytes and early embryos showed a strong punctate cytoplasmic signal that appeared to coalesce into larger aggregates as the embryos developed (Figure 1C) and these foci appeared by light microscopy to be lipid droplets. To test this hypothesis we stained mutant MATER GV oocytes (which have elevated levels of lipid droplets [18]) with Nile Red or with the H3Cit26 antibody. Results show (Additional file 1) that the H3Cit26-containing cytoplasmic foci clearly appear to be lipid droplets. Interestingly, a recent study found that lipid droplets in Drosophila embryos are maternally-derived and that these structures contain ~50% of all embryonic histones. This finding suggests that the lipid droplets function to sequester maternal histones in the early embryo until they are needed for chromatin-based activities [19]. An intriguing possibility is that the H3Cit26 modification marks histones for lipid droplet storage and/or possibly shuttling histones between lipid droplets and the nucleus. H3Cit26 staining within the nucleus was also interesting; whereas little to no signal was seen in GV stage oocytes, strong staining was observed on the outer margins of both male and female pronuclei and around the nucleoli of two-cell embryos (Figure 1C; arrows in panels of 2-PN and 2-cell). By the four-cell stage of development no nuclear staining was observed. Given that embryonic genome activation is known to initiate at the late pronuclear/early two-cell stage, this observation raises the possibility that this particular citrulline modification may play a role in activation of the embryonic genome. Taken together, our data raise the possibility that the different histone modification sites may play different roles in preimplantation development.

Given that each of these anti-citrullinated histone antibodies showed both cytoplasmic and nuclear localization patterns, we next confirmed the specificity of our antibodies by testing whether pre-absorption of the antibodies with their cognate peptide affected indirect immunofluorescence signal intensity levels (Additional file 2). Results showed that peptide preabsorption suppressed the fluorescence intensity for each of the three antibodies (Additional files 2A, 2B, and 2C, respectively). These results suggest that the localization patterns observed for the H4Cit3, H3Cit2 + 8 + 17, and H3Cit26 are specific.

### Cl-amidine blocks mouse embryonic development beyond the two to four cell stage *in vitro*

The above observations suggested that PADI-mediated histone citrullination may play an important, previously unknown, role in early development. Given that PADI6 is essential for early cleavage divisions, we next tested whether levels of these modifications were reduced in PADI6-null mouse oocytes/early embryos. We found that loss of PADI6 did not appear to affect histone citrullination levels (see Additional file 3A, 3B, and 3C). Given PADI4’s previously documented roles in histone citrullination and gene regulation [13,14], we then tested citrullinated histone levels in PADI4-null oocytes. Again, we did not observe any appreciable loss in levels of citrullinated histone in this mutant line (see Additional file 4A and 4B). Together, these observations suggest, neither PADI4 nor PADI6 catalyze these specific citrulline modifications on histones in oocytes or early embryos.

**Figure 2:**
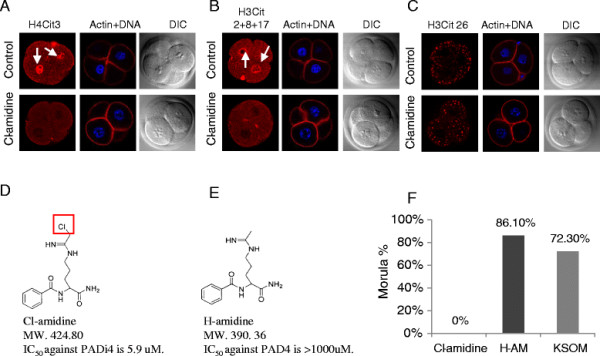
**Cl-amidine blocks embryonic development*in vitro*at the 2 to 4 cell stage. A.** Immunofluorescent confocal images showing that Cl-amidine reduces citrullination at H4R3 in 4-cell embryos. PN stage zygotes were cultured for ~42 hours or ~68 hours in KSOM medium supplemented without or with 250 μM of Cl-amidine. Four-cell embryos were fixed and immunostained with anti-H4Cit3 antibodies (red). Phalloidin and DAPI were employed to detect F-actin (red) and DNA (blue). Arrows highlight H4Cit3 staining on chromatin. DIC, differential interference contrast. **B.** Same as (A) except with anti-H3Cit2 + 8 + 17 antibodies. **C.** Same as (A) except with anti-H3Cit26 antibodies. **D.** Structure of Cl-amidine. The red square highlights the chlorine group that is replaced with hydrogen to generate H-amidine. MW., Molecular weight. **E.** Structure of H-amidine. **F.** Rate of development to the morula stage following Cl-amidine treatment. Pronuclear stage zygotes were cultured for ~68 hours in KSOM medium supplemented with 250 μM of Cl-amidine or H-amidine and observed by light microscopy. H-AM, H-amidine.

Given these observations, and the lack of mutant PADI1, PADI2, and PADI3 mouse lines, we next decided to test the effects of a newly developed PADI inhibitor, Cl-amidine, on histone citrullination and on early embryonic development. Cl-amidine has been shown to irreversibly block the activity of all PADI enzymes *in vitro* and has also been shown in cell culture and mouse-based assays to functionally inhibit PADI activity *in vivo*[20-22]. We first tested whether Cl-amidine suppressed citrulline levels on histones in early embryos using the H4Cit3, H3Cit2 + 8 + 17, and H3Cit26 antibodies. PN zygotes were cultured in KSOM media supplemented without or with 250 μM of Cl-amidine. Embryos at the 4-cell stage from KSOM and Cl-amidine groups were fixed after being cultured for ~42 hours and ~68 hours, respectively, to ensure developmental arrest at cleavage stage. Next, the embryos were stained with the anti-citrullinated histone antibodies and then evaluated by laser scanning confocal microscopy. Results showed that staining levels for the H4Cit3 and H3Cit2 + 8 + 17 antibodies was reduced, compared with the KSOM control group (Figure 2A and 2B, arrows in top panels). Interestingly, however, Cl-amidine treatment did not appear to affect levels of the H3Cit26 modification, suggesting that histone citrullination at this site may have occurred in oocytes prior to drug treatment (Figure 2C). Actin levels and localization did not appear to be affected by Cl-amidine. These results support the hypothesis that the histone citrullination in embryos is catalyzed by PADI activity.

We next investigated the effects of Cl-amidine on embryonic development *in vitro*. As a control for these experiments, we also tested the effect of H-amidine on development. This analog displays very weak PADI inhibitory activity [31] with, for example, the IC50 values of Cl-amidine and H-amidine for PAD4 inhibition *in vitro* being 5.9 μM and > 1000μM, respectively [31]. The structures of these two compounds are shown in Figure 2D and 2E. PN stage zygotes were cultured for ~68 hours in KSOM media supplemented with 250 μM of either Cl-amidine or H-amidine. The number and developmental stage of embryos was then evaluated using light microscopy. Results showed that embryos arrested either at the 2-4 cell stage (83%, n = 100) or at the 1-cell stage (17%, n = 100) in the Cl-amidine group, while 86.1% of embryos (n = 94) in the H-amidine group and 72.3% of embryos (n = 36) in KSOM medium alone developed to the morula stage (Figure 2F and Table 1). We note here that (1) the concentration of Cl-amidine used in our study is within the range of that used to functionally block PADI activity in somatic cells [14,23] and that (2) lower concentrations of Cl-amidine did not affect embryonic development (Additional file 5A and - 5B). Our finding that Cl-amidine suppressed histone citrullination in cleavage-stage embryos suggested that the observed effects of Cl-amidine on development were due to specific inhibition of PADI activity. However, it is also possible that the inhibitor blocked development because of non-specific toxic side-effects. To address this possibility, we first examined embryo viability following Cl-amidine and H-amidine treatment using the vital dye propidium iodide (PI). Results showed that nuclei from both Cl-amidine and H-amidine treated (250 μM) embryos were not stained with PI (20 μg/ml) while nuclei from embryos that were treated with Cl-amidine and extracted with 0.1% Triton were strongly stained with PI (Additional file 6A). These results indicate that the plasma membrane of Cl-amidine and H-amidine treated embryos appeared functional. To further confirm embryo viability, we next evaluated the mitochondrial membrane potential of Cl-amidine and H-Amidine treated embryos using the JC-1 fluorescent dye, which accumulates in functional mitochondria as red-staining aggregates (Additional file 6B). Results showed that the mitochondrial membrane potential appeared to be similar between the Cl-amidine and H-amidine treatment groups, suggesting the Cl-amidine does not affect mitochondrial health. Together, these findings suggest that PADI activity is required for progression of embryonic development beyond the two to four cell stage.

**Table 1:** The effect of Cl-amidine on early embryonic development

**Group**	**No.**	**Stage of embryos (%)**		
		**1 Cell**	**2-4 Cell**	**Morula**
Cl-amidine	100	17 (17)	83 (83)	0 (0)
H-amidine	36	5 (13.9)	0 (0)	31(86.1)
KSOM	94	22 (23.4)	4 (4.3)	68 (72.3)

### Treatment of embryos with C-amidine suppresses histone H3 and H4 acetylation while having no apparent effect on the repressive H3K9 Di-methyl modification

**Figure 3:**
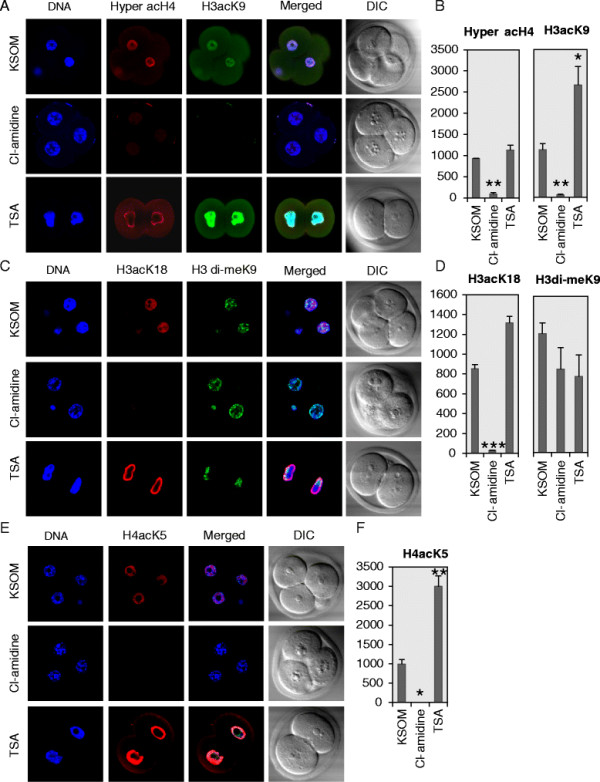
**Effect of Cl-amidine and TSA treatment on histone H3 and H4 acetylation and methylation. A.** PN zygotes were cultured for ~68 hours in KSOM medium supplemented with either 250 μM of Cl-amidine or 100 nM of TSA. Two or four-cell embryos were fixed and immunostained with antibodies to hyperacetylated H4 (red) and acetyl H3K9 (green). DAPI was utilized to detect DNA (blue). DIC, differential interference contrast. **B.** Histograms documenting the fluorescent intensity of the hyperacetylated H4 and acetyl H3K9 signals shown in A. **C.** Same as (A) except antibodies against acetyl H3K18 (red) and di-methyl H3K9 (green) were used. **D.** Histograms documenting the fluorescent intensity shown in B experiments. **E**. Same as (A) except antibodies against acetyl H4K5 (red) were used. **F.** Histogram documenting the fluorescent intensity in experiment shown in E. Data are presented as mean + SEM. *P < 0.05, **P < 0.005, ***P < 0.001 (two-tailed paired Student's *t*-test).

As noted, histone acetylation is well correlated with activation of gene expression in somatic cells and is also believed to play an important role in modulating gene expression in preimplantation embryos [7-9]. In order to begin testing whether histone citrullination may play a role regulating gene activity in early embryos, we tested whether suppression of histone citrullination with Cl-amidine affected levels of histone acetylation on H3 and H4 tails. Results showed that Cl-amidine treatment significantly reduced levels of histone H4 acetylation (Figure 3A and 3E). The fluorescent intensities for hyper acetylated H4 (hyper acH4) and H4K5 Acetyl (H4acK5) in Cl-amidine, TSA, and KSOM groups were presented in Figure 3B and 3F. Additionally, Cl-amidine also dramatically reduced the level of acetylation on the H3 tail, namely H3K9 Acetyl (H3acK9) and H3K18 Acetyl (H3acK18) (Figure 3A and 3C). The fluorescent intensities for these modifications were showed in Figure 3B and 3D. To gain further insight into the mechanisms by which histone citrullination may regulate gene activity, we also tested whether Cl-amidine affected levels of di-methylated histone H3K9, a modification closely associated with transcriptional repression [24]. We found that levels of this modification were not significantly affected by Cl-amidine treatment (Figure 3C and 3D). To validate the specificity of the acetylated histone antibodies, the embryos were also treated with the HDAC inhibitor Trichostatin A (TSA), and as expected, staining for acetylated histones (hyper acH4, H4acK5, H3acK9, and H3ack18) was elevated following TSA treatment (Figure 3). Taken together, these results suggest that inhibition of PADI-mediated histone citrullination suppresses histone acetylation.

### Histone hyperacetylation promotes histone citrullination in early embryos

**Figure 4:**
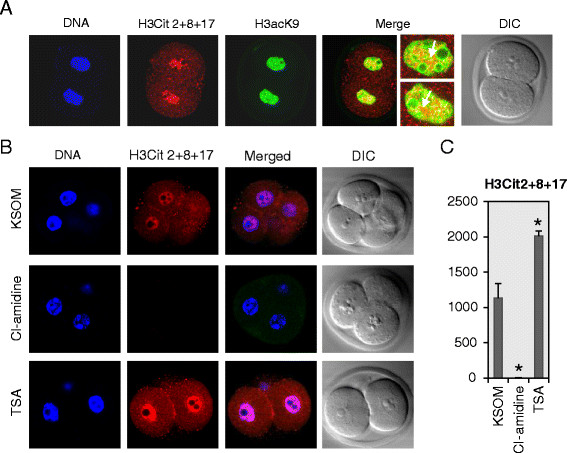
**Potential crosstalk between histone citrullination and acetylation in early embryos. A.** Colocalization of citrullinated H3R2 + 8 + 17 and acetylated H3K9. Two-cell embryos were immuno-stained with antibodies to H3Cit2 + 8 + 17 (red) and H3acK9 (green). The colocalization is highlighted in yellow in the merged panel. **B**. PN stage zygotes were co-cultured for ~68 hours in KSOM medium with 250 μM of Cl-amidine or 100 nM of TSA. Two or four-cell embryos were fixed and immuno-stained with antibodies to H3Cit 2 + 8 + 17 (red). DAPI was utilized to detect DNA (blue). DIC, differential interference contrast. Data are presented as mean + SEM. *P < 0.05, (two-tailed paired Student's *t*-test).

To further explore the potential interplay between histone citrullination and acetylation, the colocalization of citrullination at H3R2 + 8 + 17 and acetylation at H3K9 was tested in 2-cell embryos. Results revealed that these two histone modifications localize, in part, to different regions of the nucleus, and they do appear to colocalize at specific foci in 2-cell embryos (Figure 4A, arrows highlight overlapping regions which are seen as yellow in the overlay). To further test whether there was a potential crosstalk between histone citrullination and acetylation, levels of H3R2 + 8 + 17 citrullination were examined in embryos treated with either TSA or Cl-amidine. To perform this experiment, PN zygotes were recovered from B6D2F1/J females and cultured in KSOM medium supplemented with either 100 nM of TSA or 250 μM of Cl-amidine, respectively, for ~68 hours. Results showed that, as expected, levels of H3Cit2 + 18 + 17 were significantly reduced following Cl-amidine treatment (Figure 4B-C). Interestingly, however, we found that induction of histone hyperactylation via TSA treatment also resulted in significantly increased levels of citrullination at H3R2 + 8 + 17 (Figure 4B-C). This observation further suggests that there is a reinforcing relationship between acetylation and citrullination and further highlights the potential interplay between these two modifications on chromatin in preimplantation mammalian embryos.

## Conclusions

This report is the first to document the presence of citrullinated histones in mammalian oocytes and preimplantation embryos. The use of three site-specific citrullinated histone antibodies found that histone citrullination is likely playing several unique, yet to be defined roles on chromatin templated events. We found that the PADI inhibitor, Cl-amidine, potently blocks embryonic development beyond the 4-cell stage, thus further highlighting the important role of PADIs in early development. This observation also raises the possibility that PADI inhibitors could potentially be utilized as novel contraceptives. Our study also showed that Cl-amidine specifically suppressed histone acetylation on the H3 and H4 tails while not affecting levels of the transcriptionally repressive histone H3K9 dimethyl modification. Further, we found that induction of histone hyperacetylation leads to enhanced histone citrullination. Mechanistically, as with numerous other histone modifications [10,11], these observations raise the distinct possibility that the citrulline modification on histones may function as a “platform” for binding by histone acetyltransferases (HATs), thus facilitating transcriptional activation by enhancing levels of histone acetylation. More detailed studies are now required to test this hypothesis. We predict that outcomes from the current study will likely lead to new and important insight into epigenetic regulation of the oocyte to embryo transition.

## Methods

### Animals

The generation of mouse mutants and genotyping strategies for the *Padi6* and *Mater* null strain has been described previously [17,25]. To generate *Padi4*-null mice, the entire genomic sequence of *Padi4* was replaced in frame with the coding sequence of LacZ and a Lox-flanked neomycin gene driven by PGK-EM7 promoter. B6D2F1/J and CD-1 mice were purchased from the Jackson Laboratory and Charles River Laboratories, respectively. All mice were housed in the Cornell University Animal Facility (Ithaca, NY) and procedures using these mice were reviewed and approved by the Cornell University Institutional Animal Care and Use Committee. Studies were performed in accordance with the Guiding Principles for the Care and Use of Laboratory Animals.

### Oocyte and embryo collection

All experiments were performed using B6D2F1/J and CD-1 female mice (age 4-8 weeks) primed with gonadotrophins (Sigma) to obtain fully-grown GV oocytes, ovulated oocytes, and embryos (following mating with CD1 males). All oocytes and embryos were collected in M2 medium (Sigma) unless otherwise stated. Culture medium was supplemented with 25 mM of milrinone (Sigma) to inhibit GVBD. Embryos at different developmental stages were collected and processed at different times.

### Immunofluorescence and laser scanning confocal microscopy

Indirect immunofluorescence labeling confocal microscopy was undertaken as described previously [26]. Rabbit-anti-H4Cit3 (1:50, Abcam), rabbit-anti-H3Cit2 + 8 + 17 (1:50, Abcam), rabbit-anti-H3Cit26 (1:50, Abcam), rabbit-anti-hyper acetyl H4 (1:50, Millipore), mouse-anti-acetyl H3K9 (1:20, Abcam), rabbit-anti-acetyl H4K5 (1:50, Abcam), rabbit-anti-acetyl H3K18 (1:50, Abcam), and mouse-anti-dimethyl H3K9 (1:20, Abcam) antibodies were used for this study. Images were obtained on LSM 510 laser scanning confocal microscopy (Carl Zeiss, Germany) equipped with Zen 2007 software for image processing. In the multicolor labeling experiments, the confocal configuration was set up to avoid the bleed-through of fluorescence dyes. To test for bleed-through, oocytes were stained with each dye separately and images were taken with multiple channels. Each signal was found to be well resolved from other signals. Images for each developmental series were collected under similar conditions using the following settings: ex = 555 nm, em = 565 nm, laser power 12%, frame size 1024 × 1024, scanning speed 7, averaging number 4, detector gain around 700, digital offset around -15, and Zen 2009 software. To directly compare changes in signal intensity for embryos from different treatment groups, confocal images were acquired under an identical condition for those samples.

### Embryo culture and drug treatment

The KSOM medium for embryo culture has been described elsewhere [27].B6D2F1/J females were primed with 10 IU PMSG (Sigma) followed by 10 IU hCG (Sigma) injection, then housed with CD1 males. PN zygotes were collected in M2 medium at ~26 hours post hCG and cultured in KSOM for ~ 42 hours or 68 hours supplemented with 250 μM of Cl-amidine, 250 μM of H-amidine, or with 100 nM of TSA (Sigma). Embryos cultured at 37 °C in an atmosphere of 5% CO_2_, 5% O_2_ and 90% N_2_ were fixed and immunostained with antibodies at different time points for analyses. Digital images were recorded on the confocal microscopy.

### Citrulline antibody absorption assay by antigen peptide

All antibodies and antigen peptides were purchased from Abcam with the exception of the H4Cit3 peptide which was a kind gift from David Allis at Rockefeller University. The ratios of antibody and peptide for H4Cit3, H3Cit2 + 8 + 17, and H3Cit26 were 1mol:20 mols, 1μl:6 μls, and 1mol:40mols, respectively. The antibody and peptide were added to the antibody dilution buffer (1% normal goat serum, 0.5% BSA in PBS) and incubated on a rotator at room temperature for 2 hours for the H4Cit3 and H3Cit2 + 8 + 17 mixtures or for 1 hour (4°C) for the H3Cit26 mixture.

Deionized water replaced the histone modification peptides as a control. GV-oocytes or 2-cell embryos were collected from CD1 females at ~46 hours post PMSG and ~ 46 hours post hCG and processed for immunofluorescence and laser scanning confocal microscopy as described above.

### Nile red staining of mouse oocytes

Nile red powder (Sigma) was dissolved in DMSO to give a stock solution of 1mg/ml and stored at -20°C. GV-oocytes were collected in M2 media supplemented with IBMX from *Mater* mutant females. After three quick washes in PBS/PVA, oocytes were transferred into 4% paraformaldehyde/PBS and incubated for 30 min at room temperature. Oocytes were briefly washed three times in PBS/PVA again and transferred into nile red working solution (1μg/ml) for 30 min following the fixation. After the nile red staining, oocytes were washed three times and carefully added to the drop of Slowfade Gold antifade reagent (Molecular probe) on slides, and then a cover slide was placed on top of the drop. Nile red fluorescence was captured by Laser Scanning Confocal microscopy (Carl Zeiss).

### Analysis of embryo viability

Pronuclear stage zygotes were retrieved from B6D2F1/J females at ~26 hours post hCG and cultured for ~68 hours in KSOM medium supplemented with 250 μM of Cl-amidine or H-AM. Embryos from these two groups were then incubated with 20 μg/ml of PI (Sigma) in KSOM for 5 min at 37 °C in an atmosphere of 5% CO_2_, washed 3 times, and images were recorded using Zeiss epifluorescence microscopy (Carl Zeiss, Germany). A sub set of Cl-amidine treated embryos were permeablized with 0.1% Triton for 20 min prior to PI staining to serve as positive control.

JC-1 staining of cultured embryos was performed according to the Invitrogen protocol. Briefly, embryos were stained with 10 μg/ml of JC-1(Invitrogen) in KSOM for 10 min at 37 °C in an atmosphere of 5% CO_2_, washed 3 times with KSOM, and images were captured by Zeiss epifluorescence microscopy using fluorescein isothiocyanate (FITC) and tetramethyl rhodamine isothiocyanate (TRITC) filters.

### Statistical analysis

All samples used for quantification included at least four nuclei. Data were compiled and analyzed using Microsoft Excel 2007. Comparisons were made for the intensity of histone modifications in embryos cultured in KOSM media supplemented with or without Cl-amidine using unpaired *t*-test. The threshold of statistical significance was set at *P* < 0.05.

## Authors' contributions

RK participated in the design of the study, performed experiments, and wrote the paper. MJ performed the mouse embryo culture experiment with Cl-amidine. SAC conceived, designed and coordinated the study, and also helped to write the paper. VS, CPC, and PRT contributed the PADI inhibitors and helped to coordinate the study. All authors read and approved the final manuscript.

## Supplementary Material

Additional file 1 **Localization of H3Cit26 to lipid droplets in mutant*Mater*GV stage oocytes.** Mutant *Mater* oocytes (which contain high levels of lipid droplets) were probed with antibodies to H3Cit 26 (**A**) and the oocytes were also stained with Nile Red (**B**). Confocal images were taken of the stained oocytes following counterstaining with DAPI to visualize DNA (blue). DIC, differential interference contrast. Click here for file

Additional file 2 **Peptide pre-absorption assay to test for antibody specificity.** Confocal images were taken of CD1 GV-stage oocytes stained with H4Cit3 (**A**), H3Cit 2 + 8 + 17 (**B**) antibodies and 2-cell embryos stained with H3Cit26 (**C**) antibodies. In top panels, deionized water was used instead of the peptide solution as a control. In the bottom panels, anti-citrullinated histone antibodies were pre-absorbed with cognate peptides prior to staining oocytes and embryos. Oocytes/embryos were counterstained with DAPI to visualize DNA (blue). DIC, differential interference contrast. Click here for file

Additional file 3 **Comparison of citrullination levels at H3R2 + 8 + 17, H4R3, and H3R26 in PADI6 wild-type and null oocytes.** Confocal images were taken of wild-type and PADI6-null GV stage oocytes that had been probed with antibodies to H3Cit 2 + 8 + 17 (**A**), H4Cit3 (**B**), and H3Cit26 (**C**). Oocytes were counterstained with DAPI to visualize DNA (blue). DIC, differential interference contrast. Click here for file

Additional file 4 **Comparison of citrullination levels at H3R2 + 8 + 17 and H4R3 in PADI4 wild-type and null oocytes.** Confocal images were taken of wild-type and PADI4 null GV stage oocytes that had been probed with antibodies to H3Cit2 + 8 + 17 (**A**) and H4Cit3 (**B**). Oocytes were counterstained with DAPI to visualize DNA (blue). DIC, differential interference contrast. Click here for file

Additional file 5 **Titration of Cl-amidine concentration for mouse embryo culture study. A.** Pronuclear stage zygotes were cultured for ~68 hours in KSOM medium supplemented without or with 500 nM, 5 μM, or 250 μM of Cl-amidine and images of the embryos were recorded by light microscopy. Images were taken in 40X magnification with DIC and the scale bar is 50 μm. **B**. Histogram showing the rate of embryonic development for the different treatment groups in (A). (n) = total number of embryos for each group. DIC, differential interference contrast. Click here for file

Additional file 6 **Assessment of embryo viability following Cl-amidine treatment.** Pronuclear stage zygotes were cultured for ~68 hours in KSOM medium supplemented with 250 μM of Cl-amidine or H-amidine prior to the staining. **A.** Propidium iodide (PI) staining of cultured embryos. Embryos were stained with 20 μg/ml of PI in KSOM for 5 min and images were recorded by epifluorescence microscopy. Cl-amidine treated embryos were treated with 0.1% Triton for 20 min prior to PI staining and utilized as positive control to show the nuclear staining of nonviable cells. Propidium iodide, PI. **B.** JC-1 staining of embryos following treatment with Cl-amidine or H-amidine. Embryos were stained with 10 μg/ml of JC-1 in KSOM for 10 min and fluorescence was captured by epifluorescence microscopy. DIC, differential interference contrast. Click here for file
