# Supplementary figures and images for: Potential role for PADI-mediated histone citrullination in preimplantation development
Source: BMC Dev Biol. 2012 Jun 19;12:19. doi: 10.1186/1471-213X-12-19 (PMC3430579; doi:10.1186/1471-213X-12-19)

A

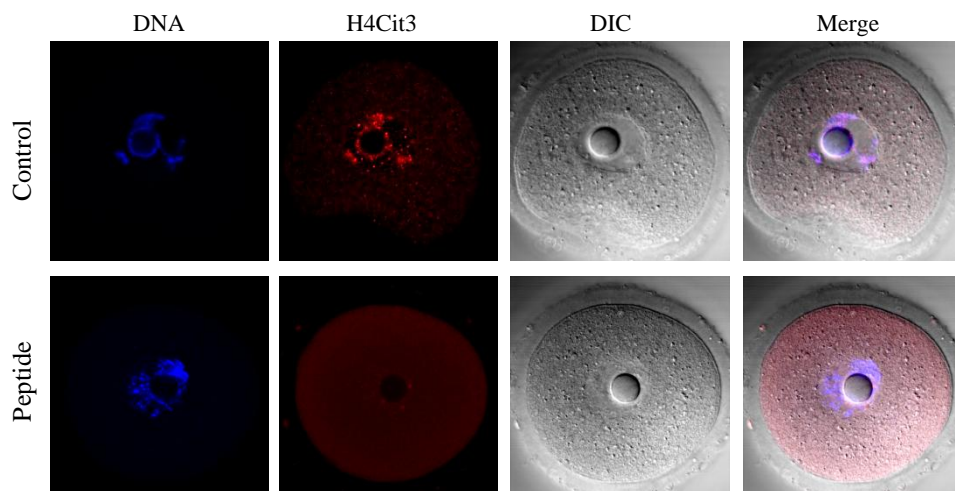

B

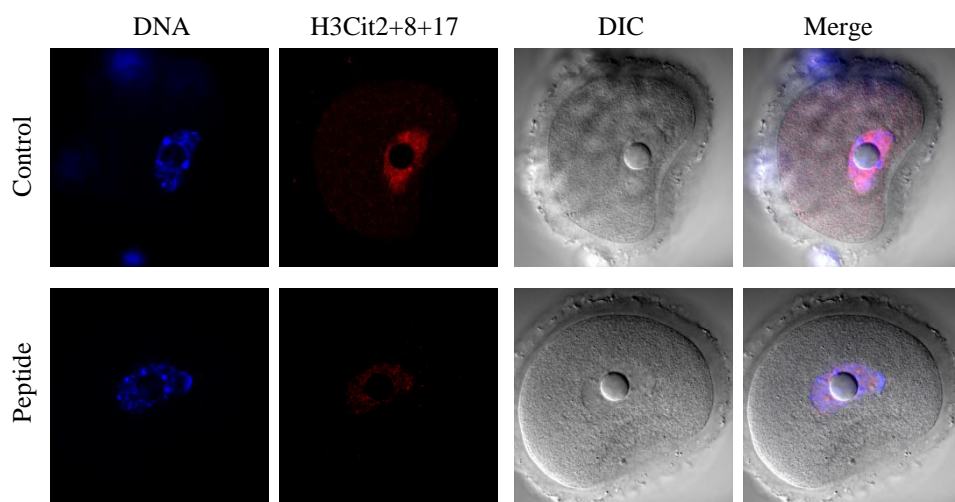

C

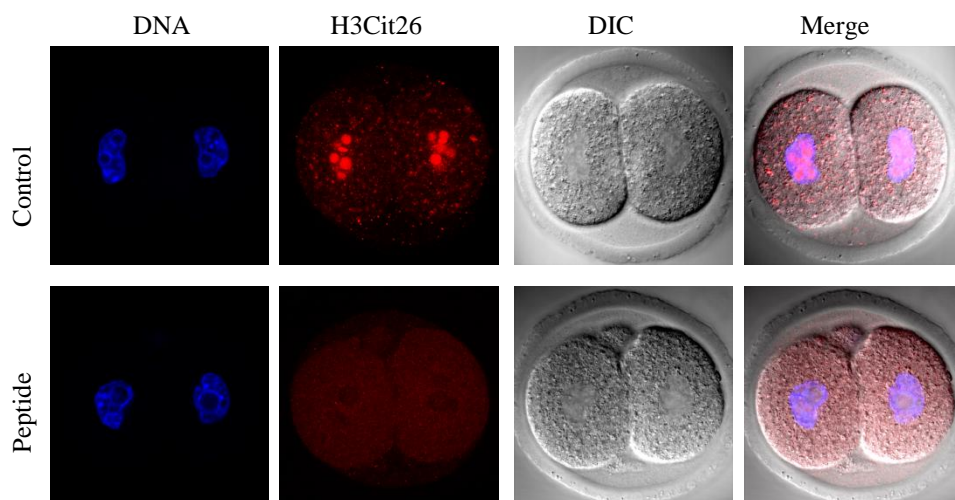

Supplement: Additional file 1 — Localization of H3Cit26 to lipid droplets in mutantMaterGV stage oocytes. Mutant Mater oocytes (which contain high levels of lipid droplets) were probed with antibodies to H3Cit 26 (A) and the oocytes were also stained with Nile Red (B). Confocal images were taken of the stained oocytes following counterstaining with DAPI to visualize DNA (blue). DIC, differential interference contrast. [file 1471-213X-12-19-S1.pdf]

A

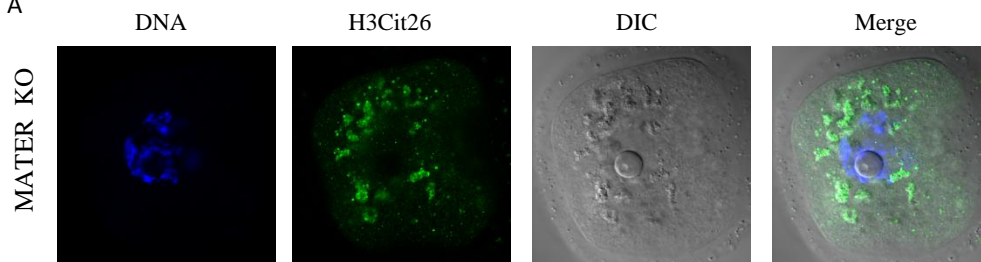

B

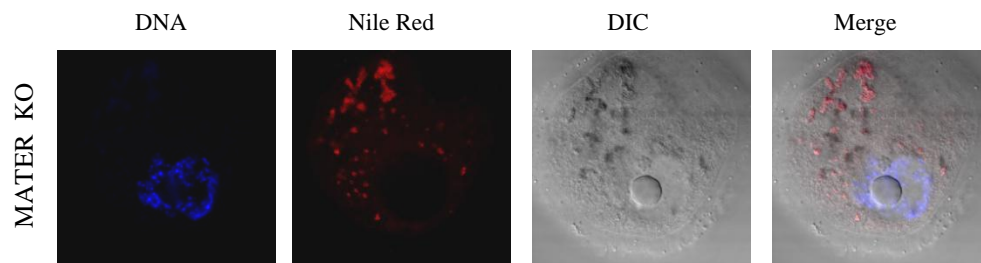

Supplement: Additional file 2 — Peptide pre-absorption assay to test for antibody specificity. Confocal images were taken of CD1 GV-stage oocytes stained with H4Cit3 (A), H3Cit 2 + 8 + 17 (B) antibodies and 2-cell embryos stained with H3Cit26 (C) antibodies. In top panels, deionized water was used instead of the peptide solution as a control. In the bottom panels, anti-citrullinated histone antibodies were pre-absorbed with cognate peptides prior to staining oocytes and embryos. Oocytes/embryos were counterstained with DAPI to visualize DNA (blue). DIC, differential interference contrast. [file 1471-213X-12-19-S2.pdf]

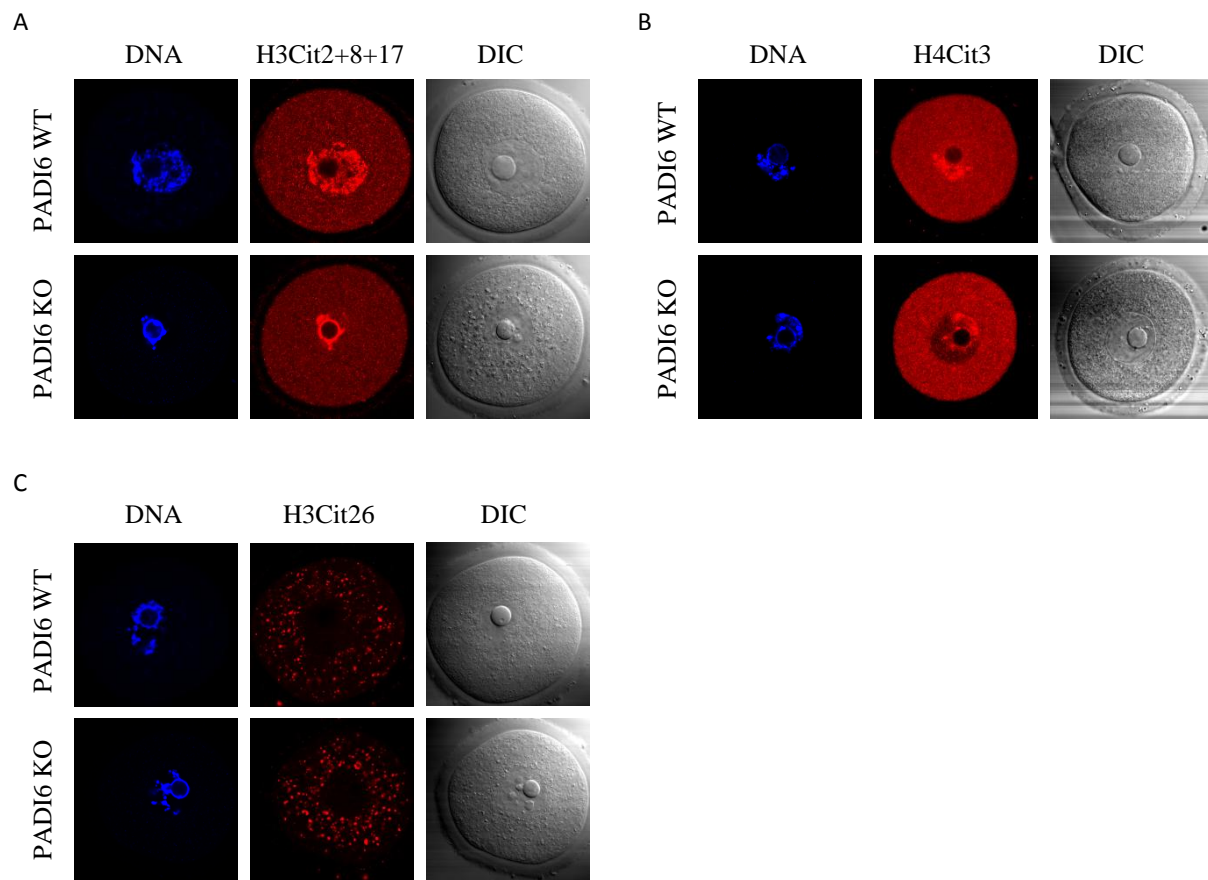

Supplement: Additional file 3 — Comparison of citrullination levels at H3R2 + 8 + 17, H4R3, and H3R26 in PADI6 wild-type and null oocytes. Confocal images were taken of wild-type and PADI6-null GV stage oocytes that had been probed with antibodies to H3Cit 2 + 8 + 17 (A), H4Cit3 (B), and H3Cit26 (C). Oocytes were counterstained with DAPI to visualize DNA (blue). DIC, differential interference contrast. [file 1471-213X-12-19-S3.pdf]

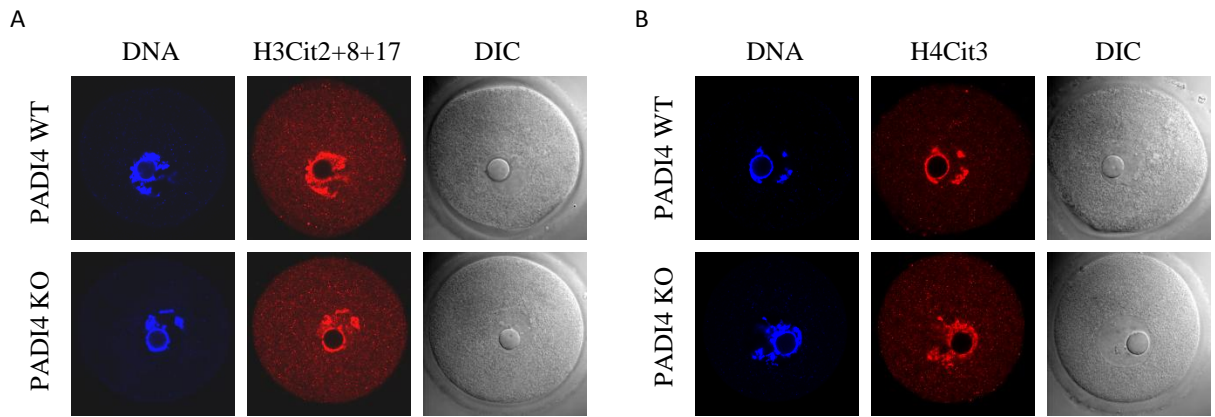

Supplement: Additional file 4 — Comparison of citrullination levels at H3R2 + 8 + 17 and H4R3 in PADI4 wild-type and null oocytes. Confocal images were taken of wild-type and PADI4 null GV stage oocytes that had been probed with antibodies to H3Cit2 + 8 + 17 (A) and H4Cit3 (B). Oocytes were counterstained with DAPI to visualize DNA (blue). DIC, differential interference contrast. [file 1471-213X-12-19-S4.pdf]

A

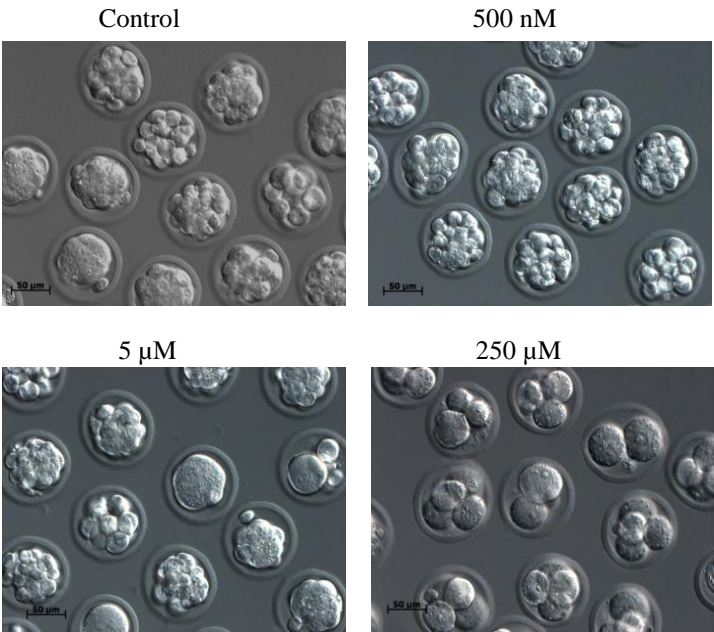

B

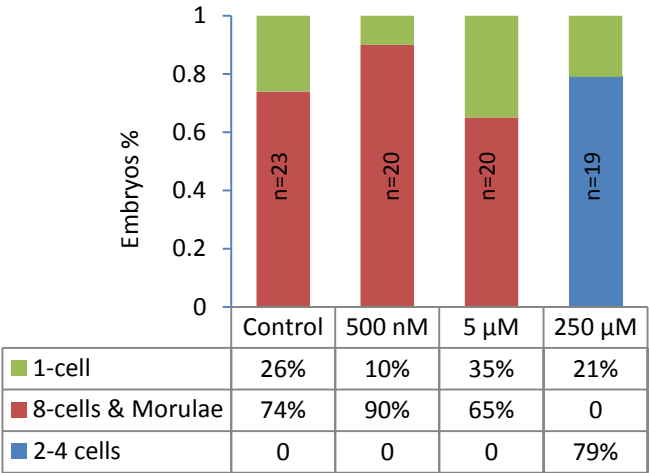

Supplement: Additional file 5 — Titration of Cl-amidine concentration for mouse embryo culture study. A. Pronuclear stage zygotes were cultured for ~68 hours in KSOM medium supplemented without or with 500 nM, 5 μM, or 250 μM of Cl-amidine and images of the embryos were recorded by light microscopy. Images were taken in 40X magnification with DIC and the scale bar is 50 μm. B. Histogram showing the rate of embryonic development for the different treatment groups in (A). (n) = total number of embryos for each group. DIC, differential interference contrast. [file 1471-213X-12-19-S5.pdf]

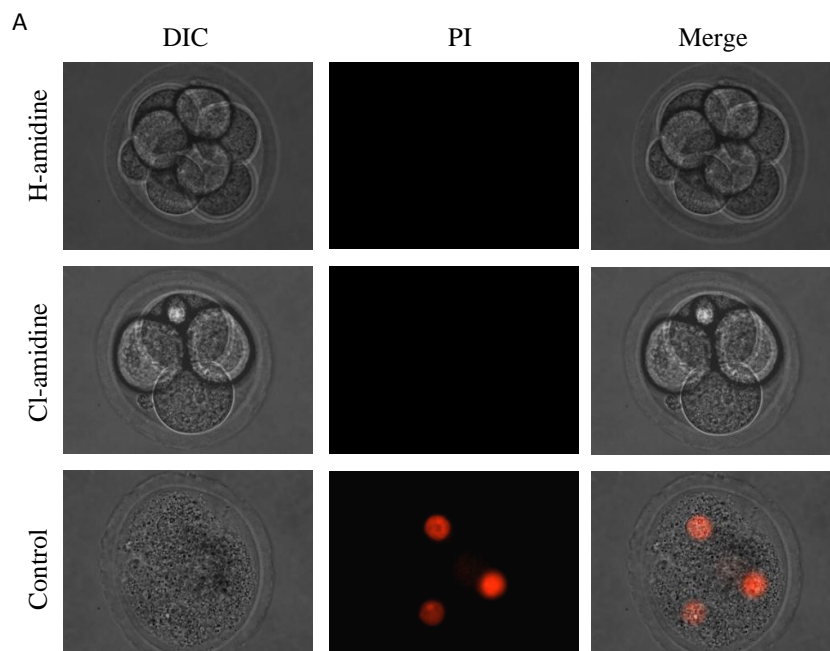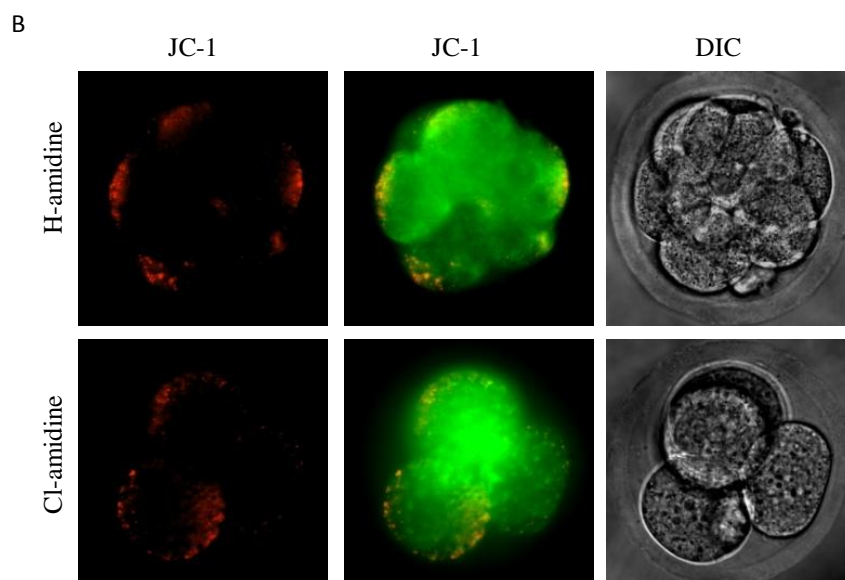

Supplement: Additional file 6 — Assessment of embryo viability following Cl-amidine treatment. Pronuclear stage zygotes were cultured for ~68 hours in KSOM medium supplemented with 250 μM of Cl-amidine or H-amidine prior to the staining. A. Propidium iodide (PI) staining of cultured embryos. Embryos were stained with 20 μg/ml of PI in KSOM for 5 min and images were recorded by epifluorescence microscopy. Cl-amidine treated embryos were treated with 0.1% Triton for 20 min prior to PI staining and utilized as positive control to show the nuclear staining of nonviable cells. Propidium iodide, PI. B. JC-1 staining of embryos following treatment with Cl-amidine or H-amidine. Embryos were stained with 10 μg/ml of JC-1 in KSOM for 10 min and fluorescence was captured by epifluorescence microscopy. DIC, differential interference contrast. [file 1471-213X-12-19-S6.pdf]
